# Color-coded circulation for visualizing swirling flow with a 3-dimensional helical stent in the superficial femoral artery

**DOI:** 10.1016/j.radcr.2024.07.089

**Published:** 2024-08-12

**Authors:** Mitsunari Maruyama, Aso Hiroya, Hisatoshi Araki, Rika Yoshida, Shinji Ando, Megumi Nakamura, Takeshi Yoshizako, Yasushi Kaji

**Affiliations:** Department of Radiology, Shimane University Faculty of Medicine, 89-1 Enya cho, Izumo-shi, Shimane, Japan

**Keywords:** Color-coded circulation, 3D helical stent, Endovascular therapy

## Abstract

Color-coded circulation is a display method that generates dynamic color-coded images based on the time of arrival of contrast agents using parametric imaging to create video displays. By cyclically displaying information in color according to the arrival time of the contrast agent at each pixel, anatomical blood vessel paths and blood flow information can be simultaneously visualized. Three-dimensional (3D) helical stents increase wall shear stress due to swirling flow and prevent intimal hyperplasia. To the best of our knowledge, there are no reports on the visualization of this swirling flow using color-coded circulation.

Here, we report the use of color-coded circulation to visualize the swirling flow following the placement of a 3D helical stent in the left superficial femoral artery. Color-coded circulation may facilitate the evaluation of contrast agent distribution and blood flow, which may otherwise go undetected with digital subtraction angiography.

## Introduction

Parametric imaging produces a color-coded static map based on the time-density curve of digital subtraction angiography (DSA) contrast media density values. Color-coded circulation is a new imaging modality based on parametric imaging, which generates videos using stepwise shifts in the color scale [1]. This color-coded circulation allows repeated visualization of the flow of contrast agents.

There is a case report indicating that color-coded circulation was useful for evaluating the vessels that were mismatched with 2-dimensional angiosome in foot perfusion during endovascular therapy [1]. However, no other reports have reported on the usefulness of color-coded circulation.

The femoropopliteal artery is long and relatively straight under resting conditions, making it difficult for swirling flow to occur and resulting in low wall shear stress [2]. This creates an environment conducive to the development of atherocslerosis. Wall shear stress exceeding 1.5 Pa inhibits the formation of atheroma, while low wall shear stress less than 0.5 Pa is associated with the development of atherosclerosis and restenosis [3].

The 3-dimensional (3D) helical stent has a spiral shape. A spiral shape increases wall shear stress owing to swirling flow and reduces intimal hyperplasia [4,5]. In addition, the 3D helical shape minimizes the risk of vascular injury and stent fracture, which are common problems with straight stents in the superficial and popliteal arteries [6].

Herein, we report the use of color-coded circulation to visualize swirling flow following the deployment of a 3D helical stent in left superficial femoral artery (SFA) occlusion.

## Case report

### Three-D helical stent placement

**Fig. 1:**
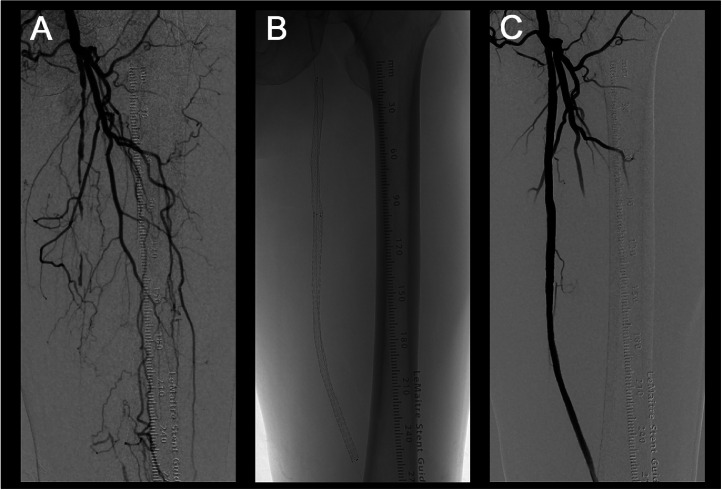
Three-dimensional (3D) helical stent placement. (A) Digital subtraction angiography (DSA) image before endovascular therapy. (B) 3D helical stent. (C) DSA image after stent placement. The stent was deployed using a 3D helical stent for left superficial femoral artery occlusion.

A 90-year-old man presented with intermittent claudication of the left lower extremity. Computed tomography angiography revealed left SFA occlusion. Endovascular therapy was performed percutaneously through antegrade puncture of the left common femoral artery. The DSA image showed left SFA occlusion (Fig. 1A). Vessel preparation was performed by the use of a balloon (ULTRASCORE™ Scoring PTA balloon catheter), and stent deployment (Fig. 1B) was carried out using a 3D helical stent. A stent-in-stent technique was employed and stent placement was successful (Fig. 1C), with the left ankle-brachial index increasing from 0.54 to 0.78.

### Color-coded circulation and 4-dimensional (4D) flow magnetic resonance imaging (MRI)

Endovascular therapy was performed using an X-ray angiography system (Alphenix Sky). Color-coded circulation is a display method that shows static color-coded images based on the time of arrival of contrast agents through parametric imaging and presents them as videos on a workstation (Alphenix WorkStation Pro). By cyclically displaying information in color according to the arrival time of the contrast agent at each pixel, anatomical blood vessel paths and blood flow information can be simultaneously depicted. The DSA images were displayed using color-coded circulations when appropriate.

A streamlined image was obtained via 4D flow MRI using a 3-T MRI scanner (Ingenia Elition X) and an image analysis system (SYNAPSE VINCENT). Four-D flow MRI of the femur was performed using an electrocardiogram-gated, time-resolved, 3D phase-contrast MR sequence with 3-directional velocity encoding. The parameters were as follows: repetition time/echo time/flip angle of 6.4 ms/4.6 ms/12°, k-space segmentation/temporal resolution of 9.3 ms/51.5 ms, field of view of 380 × 380 mm, slice thickness of 3.0 mm, and voxel size of 1.83 × 3.26 × 3.00 mm³. The acquisition took approximately 3.08 min, producing 11 timeframes per R-R interval.

**Fig. 2:**
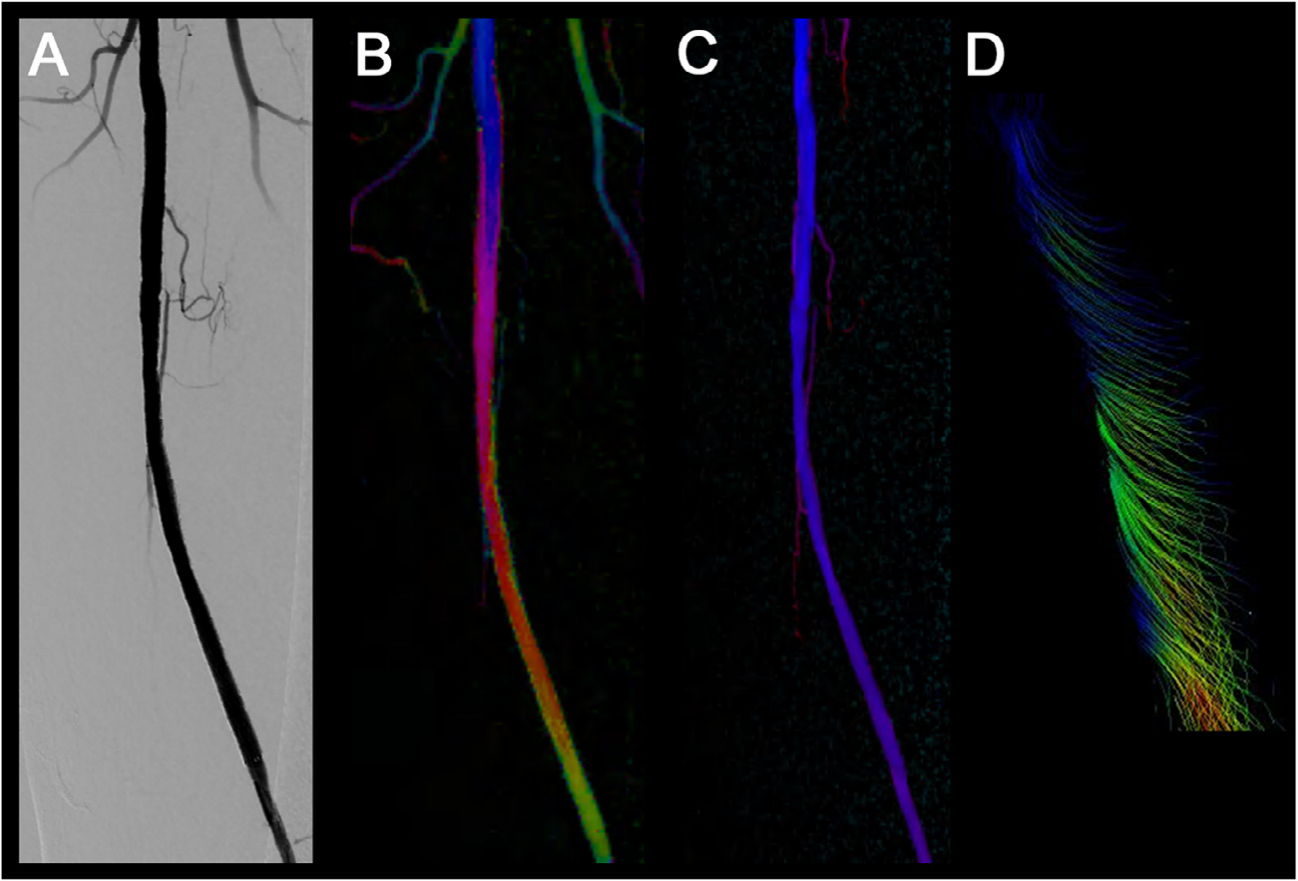
Color-coded circulation image before and after stent placement. (A) Digital subtraction angiography (DSA) image after stent placement. (B) Color-coded circulation image after stent placement. (C) Color-coded circulation image before stent placement. (D) Streamline image by 4-dimensional flow magnetic resonance imaging at the stent exit site. It was difficult to confirm the presence of swirling flow using DSA (A). Layered colors are observed at the stent site (B, supplementary material: video displays) but are not present before stent placement (C). Swirling flow is visible in the streamlined image (D).

**Fig. 3:**
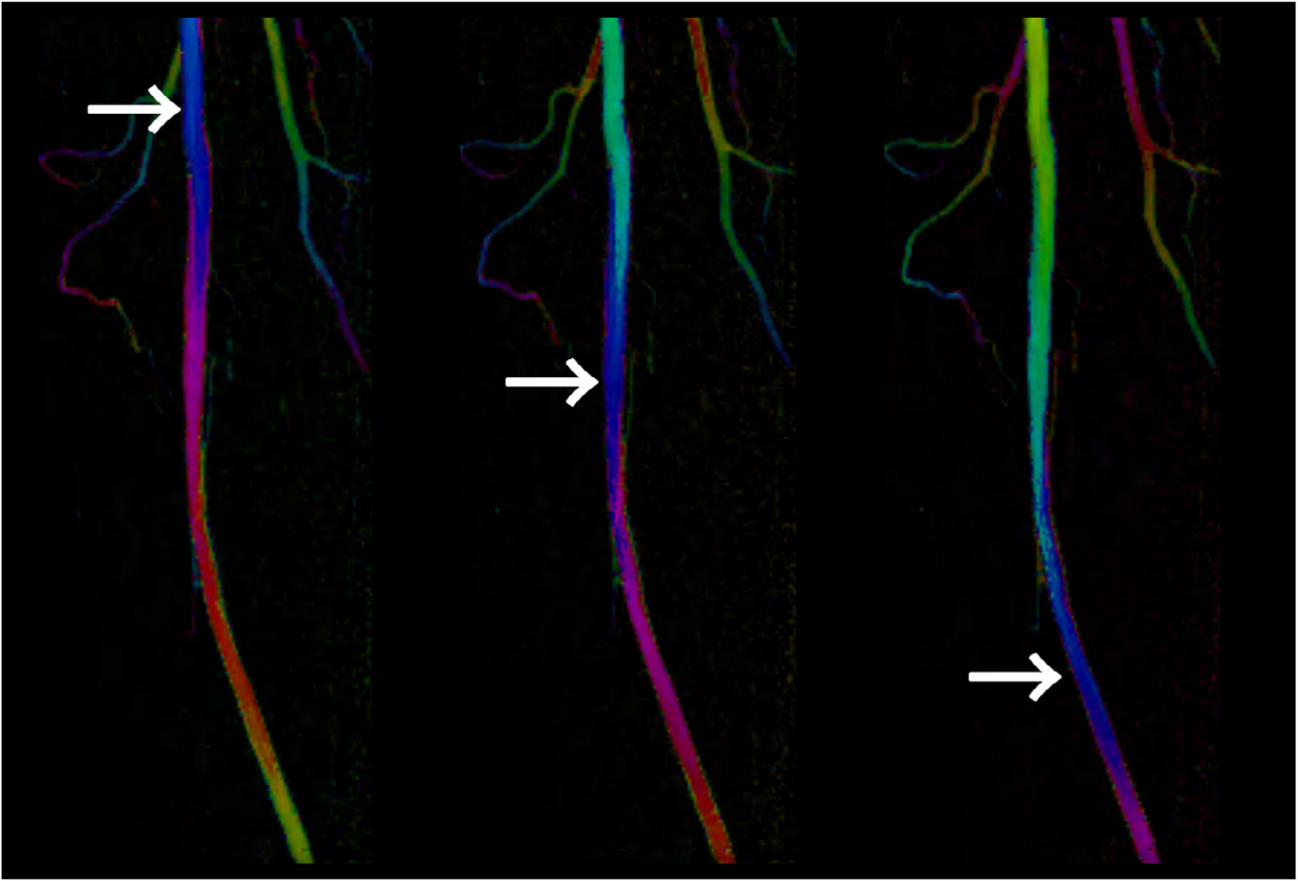
Color-coded circulation image after stent placement. Focusing on a single color, such as blue, reveals a spiral flow pattern (white arrow, supplementary material: video displays).

It was difficult to confirm the swirling flow in the DSA image (Fig. 2A) after stent placement. Layered colors were observed at the stent site (Fig. 2B, supplementary material: video displays); these were not present before stent placement (Fig. 2C). When 4D flow MRI of the stent exit site was performed later, swirling flow was observed on the streamlined image (Fig. 2D). Focusing on a single color, such as blue, revealed a spiral flow pattern (Fig. 3, white arrow, supplementary material: video displays).

## Discussion

The swirling flow in the BioMimics 3D vascular stent was visualized using color-coded circulation. Focusing on a single color revealed a spiral flow pattern. The layered colors in the images highlight the swirling flow within the stent. This color-coding technique facilitates the observation of contrast agents and blood flow, which are not easily detectable with DSA, by displaying them in distinct colors.

The BioMimics 3D stent is a 3D spiral-shaped device that creates a swirling flow within the stent. This design uses wall shear stress to promote long-term patency. When wall shear stress is applied, the vascular endothelial cells are not activated and do not secrete substances that promote arteriosclerosis, thereby suppressing intimal thickening and restenosis [5]. MIMICS-3D is a real-world European registry. After propensity score matching, clinically driven target lesion revascularization and primary patency with a 3D helical stent might not be influenced by lesion background, including chronic total occlusion, chronic limb-threatening ischemia, or highly calcified lesions [7]. The MIMICS 3D registry also demonstrated satisfactory results, comparable to drug-eluting stents, in patients with 3D helical stents [8].

## Conclusion

We report the use of color-coded circulation to visualize the swirling flow following the placement of a 3D helical stent in the left SFA. Color-coded circulation may facilitate the evaluation of contrast agent distribution and blood flow, which may otherwise go undetected with DSA.

## Patient consent

Informed consent was obtained for the publication of this case report.

## Author contributions

All authors have made significant contributions to the manuscript and approved the final version for publication.

## Appendix Supplementary materials

Image, video 1

Image, application 2
