# Supplementary material for: Color-coded circulation for visualizing swirling flow with a 3-dimensional helical stent in the superficial femoral artery
Source: Radiol Case Rep. 2024 Aug 12;19(11):4814–7. doi: 10.1016/j.radcr.2024.07.089 (PMC11367534; doi:10.1016/j.radcr.2024.07.089)
Supplement: Supplementary file 2 [file mmc2.pdf]

Certificate of Elsevier Language Editing Services

The following article was edited by Elsevier Language Editing Services:

Color-Coded Circulation for Visualizing Swirling Flow with a 3D Helical Stent in the Superficial Femoral Artery

Ordered by:

Mitsunari Maruyama

Estimated Delivery date:

2024-07-09

Order reference:

ASLESTD1066133
